# Supplementary material for: Identification of a novel PPARβ/δ/miR‐21‐3p axis in UV‐induced skin inflammation
Source: EMBO Mol Med. 2016 Jun 1;8(8):919–36. doi: 10.15252/emmm.201505384 (PMC4967944; doi:10.15252/emmm.201505384)
Supplement: Supplementary file 1 — Appendix [file EMMM-8-919-s001.pdf]

Appendix table of content:

Appendix Table S1

Appendix Table S2

Appendix Figure S1

Appendix Figure S2

Appendix Methods

Appendix Legends

Appendix Methods

miR-21-3p putative targets in silico analysis: Predictions of seed sequences were obtained from miRmap (Vejnar et al, 2013). Genomic distances of the first nucleotide of SMAD7 seed sequence predicted to bind with miR-21-3p are: sequence A 146446307 (chromosome 18) 1435 (3'UTR) and sequence B 46446291 (chromosome 18) 1451(3'UTR)

Appendix Legends

Appendix Table S1: PPARβ/δ regulates the expression of miRNAs in murine skin chronically exposed to UV

Cutaneous miRNA, which expression was affected by chronic UV exposure (12 weeks) in a PPARβ/δ-dependent way. Black: miRNA overexpressed (at least 1.5 fold) in Ppard +/+ compared to Ppard -/- skin. Light grey: miRNA underexpressed (at least 1.5 fold) in Ppard +/+ compared to Ppard -/- skin. Dark grey: miRNA underexpressed (at least 3 fold) in Ppard +/+ compared to Ppard -/- skin. Statistical significance was set to p-value < 0.001 (t-test analysis adjusted for multiple comparison).

Appendix Table S2: Identification of putative targets of miR-21-3p

Putative targets of miR-21-3p were identified by in silico analysis of miRNA Recognition Elements using the miRmap interface (Vejnar et al, 2013). miTG score: weighted sum of the scores of the miRNA Recognition Elements identified on the same 3'UTR. Putative targets with a miTG score of 0.7 or above are listed.

Appendix Figure S1: (**A**) miR-21-3p/miR-21-5p ratio: number of miR-21-3p reads on the number of miR-21-5p reads obtained from RNA sequencing in mouse brain, heart, kidney,

testis and total skin. **(B)** Normalized miR-21-3p counts from RNA sequencing in acutely irradiated (Ac-UV; +) and non-irradiated (-) epidermis and dermis of Ppard +/+ and -/- mice. miR-21-3p: Epidermis no UV vs Ac-UV P = 0.043, N=4 animals per group. (**C**) RT-qPCR quantification of Collagen IV (Col4a1, dermis), Involucrin (Ivl, supra-basal epidermis), and Keratin 5 (Krt5, basal epidermis) mRNA in mouse isolated dermis and epidermis, N=3 animals per group Ivl: Dermis vs Epidermis P = 0.046; Krt 5: Dermis vs Epidermis P = 2E-04. (**D**) RT-qPCR quantification of relative Ppard mRNA expression in epidermis and dermis of Ppard +/+ and -/- mice, acutely UV-irradiated (Ac-UV; +) or non-irradiated (-). Ppard: Epidermis no UV vs Ac-UV P = 0.007. N=4 animals per group. (**E**) RT-qPCR quantification of ANGPTL4 and TGFB1 mRNA expression in HaCaT cells treated with the PPARβ/δ agonist GW501516 or vehicle. ANGPTL4: Veh vs GW501516 P = 8E-04; TGFB1: Veh vs GW501516 P = 0.017 N=3 biological replicates. (**F**) RT-qPCR quantification of relative SERPINE1 levels in HaCaT cells treated for 24h with 2 or 5ng/ml of recombinant human TGFβ-1 or vehicle. SERPINE1: TGFβ-1 Veh vs 5ng/ml P = 0.001. N=3 biological replicates. (**G)** RT-qPCR quantification of relative ANGPTL4 and SERPINE1 expression in HaCat cells treated during 24h with the PPARβ/δ agonist GW0742 and/or the TGFβ receptor inhibitor SB431542 or vehicle as indicated. ANGPTL4: Veh vs GW0742 P = 6E-08, GW0742 vs SB431542 P = 0.002; SB431542 vs GW0742/SB431542 P = 0.033; SERPINE1: Veh vs GW0742 P = 0.039, GW0742 vs SB431542 P = 0.001, N=3 biological replicates. (**H**) RT-qPCR quantification of relative Serpine1 expression in Ppard +/+ epidermis of acutely UV-irradiated (Ac-UV; +) or in non-irradiated (-) mice treated with the TGFβ receptor inhibitor SB431542 or vehicle. Serpine1: no UV vs Ac-UV P = 1E-04, Ac-UV vs Ac-UV/SB431542 P = 7E-04, N=4 animals per group.

Appendix Figure S2: (**A**) RT-qPCR quantification of relative miR-21-3p and miR-21-5p levels in HaCaT cells transfected with a miR-21-3p mimic or a scrambled sequence (control). miR-21-3p: control vs miR-21-3p mimic P = 1E-04, N=3 biological replicates. (**B**) RT-qPCR quantification of relative miR-21-3p levels in normal mouse skin, DMBA/TPA-induced

papilloma and squamous cell carcinomas (SCC). miR-21-3p: Normal mouse skin vs Mouse skin papilloma P = 0.028, Normal mouse skin vs Mouse skin SCC P = 0.008, N=6 animals per group. (**C**) RT-qPCR quantification of relative miR-21-5p and miR-21-3p levels in normal mouse skin (control) or after gentle epidermal barrier disruption (Tape Stripping) (**D**) Fluorescent miR-21-3p or scrambled control *in situ* hybridization (pink) in healthy human skin. E: epidermis; D: dermis. Magnification bar: 20μm (**E**) RT-qPCR quantification of relative mRNA expression of PPARD and TGFB1 in healthy human skin and in human squamous cell carcinomas (SCC). PPARD: Healthy skin vs SCC P = 0.034*;* TGFB1: Healthy skin vs SCC P = 0.012. ≥ 5 independent biopsies per condition. (**F)** RT-qPCR quantification of relative mRNA expression of PPARD and TGFB1 in healthy human skin and in human psoriasis lesions. PPARD: Healthy skin vs psoriasis lesions P = 0.032*;* TGFB1: Healthy skin vs psoriasis lesions P = 0.021. ≥4 independent biopsies per condition (**G**) RT-qPCR quantification of miR-21-3p (Left panel) and TGFB1 (Right panel) in abdominal healthy human skin biopsies exposed to acute UV *ex vivo*, with or without topical treatment with miR-21-3p inhibitor (+) or mismatched control (-) as indicated. miR-21-3p: no UV vs Ac-UV P = 0.027, Ac-UV vs Ac-UV/miR-21-3p inhibitor P = 0.010. ≥ 7 biological replicates, a pool of three independent experiment performed with the skin of three different donors is shown.

miR-93-3p(*)	
miR-34c-5p	
miR-337-3p	
miR-154-5p	
miR-21-3p(*)	
miR-379-5p	
miR-299a-5p(*)	
miR-300-3p	
miR-329-3p	
miR-127-3p	
miR-434-5p	
miR-667-3p	

Ppard +/+ versus -/- Fold change (p-value < 0.001)	
	> 1.5
	< -1.5
	-3

Appendix Table S1
PPARβ/δ regulates the expression of miRNAs in murine skin chronically exposed to UV.

Appendix Table S2

miR-21-3p in silico predicted targets

Ensembl ID	Gene Id (name)	miTG score	Biological process
ENST00000253807	ENSG00000081842 (PCDHA6)	0.974	cell adhesion_homophilic cell adhesion via plasma membrane adhesion molecules
ENST00000231004	ENSG00000113083 (LOX)	0.959	blood circulation_cell adhesion_inflammatory response
ENST00000257905	ENSG00000135447 (PPP1R1A)	0.958	glycogen metabolic process_intracellular signal transduction_negative regulation of protein kinase activity
ENST00000261349	ENSG00000070018 (LRP6)	0.95	canonical Wnt signaling pathway_cerebral cortex development_face morphogenesis
ENST00000240364	ENSG00000121104 (FAM117A)	0.94	na
ENST00000220822	ENSG00000104381 (GDAP1)	0.929	glutathione metabolic process
ENST00000194672	ENSG00000082269 (FAM135A)	0.923	activation of MAPK activity
ENST00000229330	ENSG00000111727 (HCFC2)	0.921	negative regulation of transcription from RNA polymerase II promoter_viral process
ENST00000257192	ENSG00000134760 (DSG1)	0.905	apoptotic process_cell-cell junction assembly
ENST00000229769	ENSG00000112039 (FANCE)	0.905	DNA damage_DNA repair
ENST00000238831	ENSG00000119820 (YIPF4)	0.872	maintenance of the Golgi structure
ENST00000263239	ENSG00000088205 (DDX18)	0.862	RNA secondary structure unwinding
ENST00000261427	ENSG00000078140 (UBE2K)	0.857	cellular response to interferon-beta_proteasome-mediated ubiquitin-dependent protein catabolic process
ENST00000260777	ENSG00000187164 (KIAA1598)	0.856	actin filament bundle retrograde transport_axon guidance_
ENST00000256509	ENSG00000134121 (CHL1)	0.84	cellular protein metabolic process_endoplasmic reticulum unfolded protein response_IRE1-mediated unfolded protein response
ENST00000246229	ENSG00000126003 (PLAGL2)	0.84	chylomicron assembly_lipid metabolic process_positive regulation of intrinsic apoptotic signaling pathway
ENST00000250263	ENSG00000104626 (ERI1)	0.829	DNA catabolic process, exonucleolytic
ENST00000239367	ENSG00000120256 (LRP11)	0.824	multicellular organismal response to stress
ENST00000218099	ENSG00000101981 (F9)	0.822	blood coagulation_Hemostasis
ENST00000240055	ENSG00000120837 (NFYB)	0.82	positive regulation of transcription, DNA-templated
ENST00000262052	ENSG00000110911 (SLC11A2)	0.815	cation transmembrane transport
ENST00000262124	ENSG00000085415 (SEH1L)	0.811	cell division_cytokine production involved in inflammatory response
ENST00000244745	ENSG00000124766 (SOX4)	0.805	ascending aorta morphogenesis_canonical Wnt signaling pathway_cellular response to glucose stimulus_DNA damage response, detection of DNA damage
ENST00000256682	ENSG00000134287 (ARF3)	0.804	phosphatidylinositol biosynthetic process_phospholipid metabolic process_protein transport
ENST00000251108	ENSG00000102543 (CDADC1)	0.801	na
ENST00000261226	ENSG00000057704 (TMCC3)	0.797	na
ENST00000238616	ENSG00000119638 (NEK9)	0.786	cell division
ENST00000255416	ENSG00000133055 (MYBPH)	0.777	cell adhesion
ENST00000252512	ENSG00000130227 (XPO7)	0.77	mRNA transport
ENST00000235382	ENSG00000116741 (RGS2)	0.766	brown fat cell differentiation_cell cycle_negative regulation of cAMP-mediated signaling
ENST00000227451	ENSG00000110042 (DTX4)	0.766	Notch signaling pathway_protein ubiquitination
ENST00000250448	ENSG00000129514 (FOXA1)	0.763	anatomical structure morphogenesis_cell differentiation
ENST00000251020	ENSG00000103449 (SALL1)	0.762	adrenal gland development_branching involved in ureteric bud morphogenesis_embryonic digestive tract development
ENST00000216416	ENSG00000100528 (CNIH)	0.751	immune response

ENST00000262460	ENSG00000101003 (GINS1)	0.748	DNA strand elongation involved in DNA replication
ENST00000249064	ENSG00000159873 (CCDC117)	0.748	na
ENST00000262158	ENSG00000101665 (SMAD7)	0.747	BMP signaling pathway_intracellular signal transduction_negative regulation of transforming growth factor beta receptor signaling pathway
ENST00000256649	ENSG00000134253 (TRIM45)	0.747	bone development
ENST00000261530	ENSG00000089916 (C14orf118)	0.735	na
ENST00000233969	ENSG00000115616 (SLC9A2)	0.734	ion transport_protein localization
ENST00000242208	ENSG00000122641 (INHBA)	0.732	activin receptor signaling pathway_cell cycle arrest_hair follicle development
ENST00000249377	ENSG00000128606 (LRRC17)	0.732	bone marrow development
ENST00000237853	ENSG00000118985 (ELL2)	0.731	regulation of transcription, DNA-templated
ENST00000262367	ENSG00000005339 (CREBBP)	0.73	cell proliferation_cellular lipid metabolic process_innate immune response
ENST00000261211	ENSG00000059758 (CDK17)	0.729	protein phosphorylation
ENST00000215832	ENSG00000100030 (MAPK1)	0.724	na
ENST00000263026	ENSG00000103319 (EEF2K)	0.723	insulin receptor signaling pathway_regulation of protein autophosphorylation
ENST00000242505	ENSG00000138286 (FAM149B1)	0.72	na
ENST00000255380	ENSG00000133019 (CHRM3)	0.719	adenylate cyclase-inhibiting G-protein coupled acetylcholine receptor signaling pathway_cell proliferation_energy reserve metabolic process
ENST00000258646	ENSG00000136144 (RCBTB1)	0.716	cell cycle_chromatin modification_regulation of transcription
ENST00000005257	ENSG00000006451 (RALA)	0.714	actin cytoskeleton reorganization_chemotaxis
ENST00000216252	ENSG00000100410 (PHF5A)	0.714	mRNA processing
ENST00000258198	ENSG00000135720 (DYNC1LI2)	0.709	antigen processing and presentation of exogenous peptide antigen via MHC class II_centrosome localization_microtubule-based movement
ENST00000257696	ENSG00000135245 (HILPDA)	0.709	autocrine signaling_positive regulation of cell proliferation_positive regulation of cytokine production_positive regulation of lipid storage
ENST00000261537	ENSG00000101752 (MIB1)	0.707	blood vessel development_endocytosis_Notch signaling pathway
ENST00000238647	ENSG00000119669 (IRF2BPL)	0.704	development of secondary female sexual characteristics_negative regulation of transcription from RNA polymerase II promoter
ENST00000066544	ENSG00000004897 (CDC27)	0.703	anaphase-promoting complex-dependent proteasomal ubiquitin-dependent protein catabolic process_cell proliferation
ENST00000248450	ENSG00000127837 (AAMP)	0.702	angiogenesis
ENST00000223951	ENSG00000107105 (ELAVL2)	0.702	regulation of transcription_DNA-templated
ENST00000263681	ENSG00000077514 (POLD3)	0.7	DNA damage response, detection of DNA damage

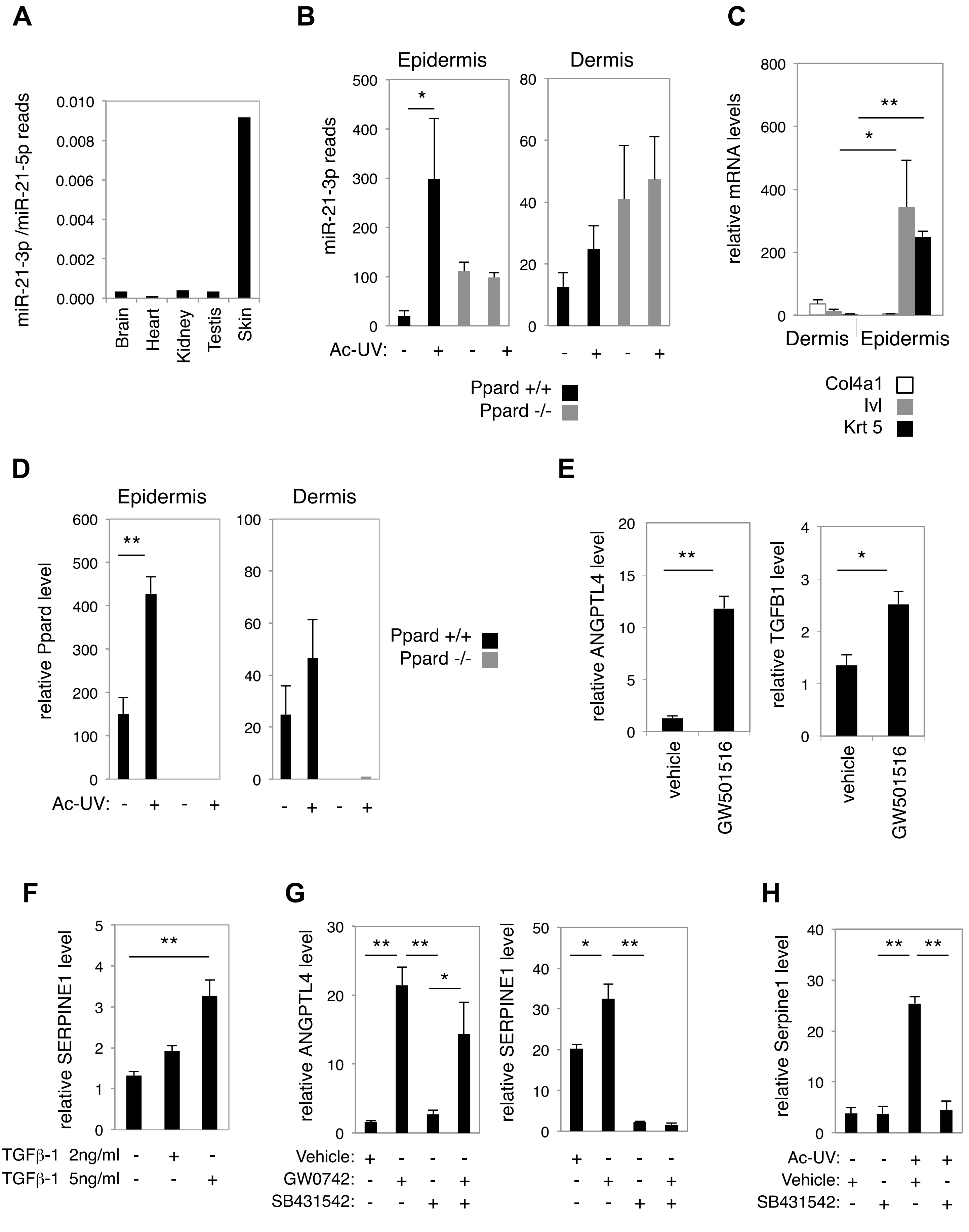

Appendix Figure S1

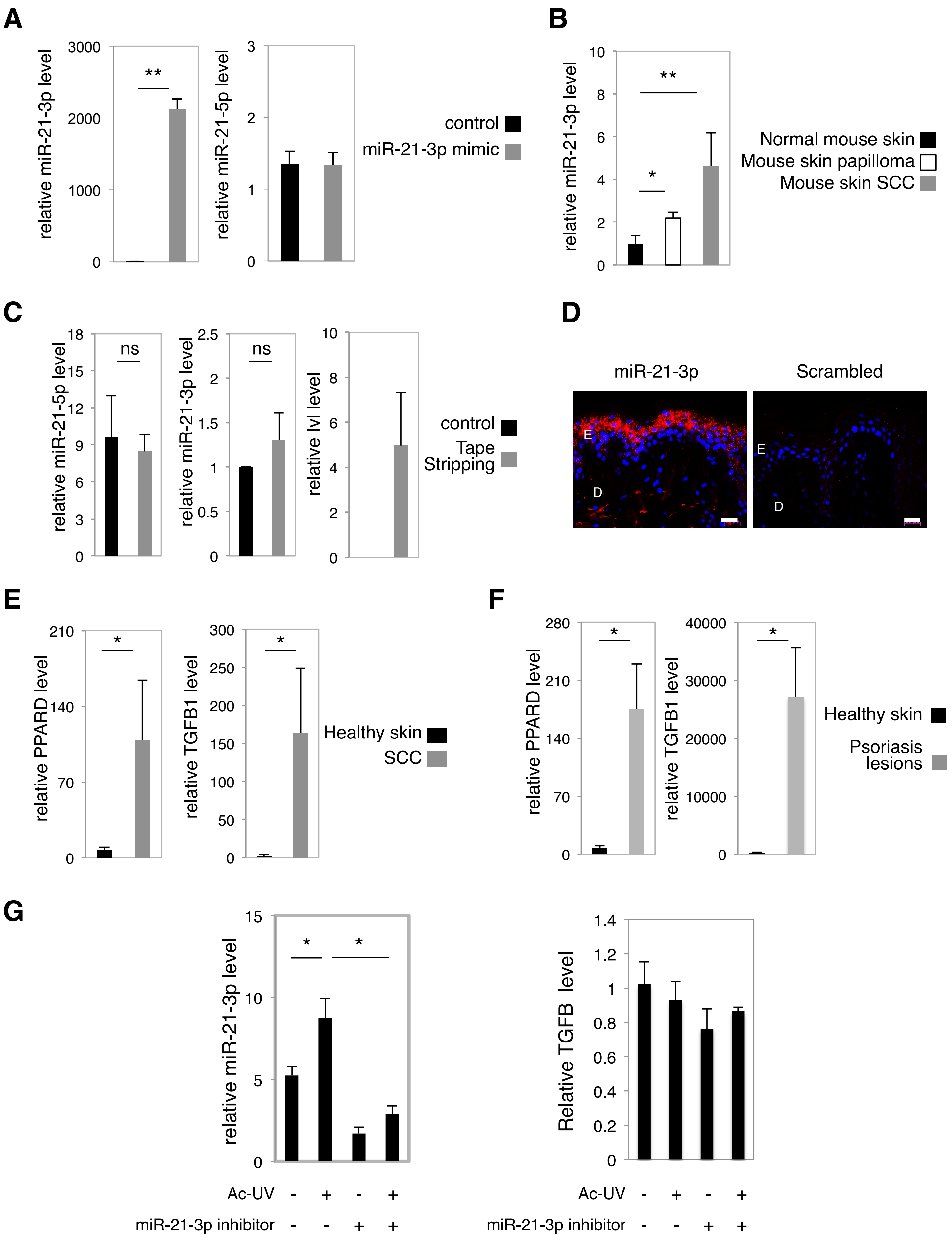

Appendix Figure S2
